# A database of virtual healthy subjects to assess the accuracy of foot-to-foot pulse wave velocities for estimation of aortic stiffness

**DOI:** 10.1152/ajpheart.00175.2015

**Published:** 2015-06-08

**Authors:** Marie Willemet, Phil Chowienczyk, Jordi Alastruey

**Affiliations:** ^1^Division of Imaging Sciences and Biomedical Engineering, St. Thomas' Hospital, King's College London, London, United Kingdom; and; ^2^Department of Clinical Pharmacology, St Thomas' Hospital, King's College London, London, United Kingdom

**Keywords:** brachial-ankle PWV, carotid-femoral PWV, numerical 1D model, aortic stiffness, database of virtual subjects

## Abstract

While central (carotid-femoral) foot-to-foot pulse wave velocity (PWV) is considered to be the gold standard for the estimation of aortic arterial stiffness, peripheral foot-to-foot PWV (brachial-ankle, femoral-ankle, and carotid-radial) are being studied as substitutes of this central measurement. We present a novel methodology to assess theoretically these computed indexes and the hemodynamics mechanisms relating them. We created a database of 3,325 virtual healthy adult subjects using a validated one-dimensional model of the arterial hemodynamics, with cardiac and arterial parameters varied within physiological healthy ranges. For each virtual subject, foot-to-foot PWV was computed from numerical pressure waveforms at the same locations where clinical measurements are commonly taken. Our numerical results confirm clinical observations: *1*) carotid-femoral PWV is a good indicator of aortic stiffness and correlates well with aortic PWV; *2*) brachial-ankle PWV overestimates aortic PWV and is related to the stiffness and geometry of both elastic and muscular arteries; and *3*) muscular PWV (carotid-radial, femoral-ankle) does not capture the stiffening of the aorta and should therefore not be used as a surrogate for aortic stiffness. In addition, our analysis highlights that the foot-to-foot PWV algorithm is sensitive to the presence of reflected waves in late diastole, which introduce errors in the PWV estimates. In this study, we have created a database of virtual healthy subjects, which can be used to assess theoretically the efficiency of physiological indexes based on pulse wave analysis.

## Abstract (precis)

*This work presents a new methodology for the theoretical assessment of computed physiological indexes and algorithms based on pulse wave analysis. We created a database of virtual healthy subjects using a 1D model of the arterial hemodynamics. This study presents its application to central and peripheral foot-to-foot pulse wave velocities*.

## NEW & NOTEWORTHY

*This work presents a new methodology for the theoretical assessment of computed physiological indexes and algorithms based on pulse wave analysis. We created a database of virtual healthy subjects using a 1D model of the arterial hemodynamics. This study presents its application to central and peripheral foot-to-foot pulse wave velocities*.

aortic stiffness has proven to be an important indicator of cardiovascular events (26). In clinical practice, it is evaluated by the carotid-femoral foot-to-foot pulse wave velocity (PWV; cfPWV). Because of its large association with cardiovascular events (10, 25, 52), its noninvasiveness, and its relative ease in determination, this index is considered the gold standard method by clinical experts (26). Reference and normal values for cfPWV have been established based on the analysis of more than 11,000 subjects (47), showing that cfPWV increases with age and blood pressure in normotensive subjects.

In recent years, the option of using the peripheral brachial-ankle PWV (baPWV) as a substitute to the central cfPWV is being studied, in particular in East Asian countries. Thanks to the use of peripheral pressure cuffs, the acquisition protocol is automatized and simplified and causes less discomfort for the subjects (43, 46). However, this index represents an average of arterial stiffness over a long arterial path, which encompasses both elastic (aorta) and muscular (femoral and brachial) arteries. As these arteries show different stiffness responses (51), baPWV might not be an accurate surrogate of aortic stiffness.

Recent studies have tried to clarify the relation between cfPWV and baPWV (23, 43, 46, 55). Within East Asian populations, results seem to converge to a strong correlation between both indexes, with baPWV overestimating cfPWV by 20% on average (46), suggesting that portions of baPWV may be determined by peripheral (muscular) arterial stiffness. Choo et al. (15) confirmed as well that there is a moderate correlation between baPWV and both heart-femoral PWV and femoral-ankle PWV. In their meta-analysis, Vlachopoulos et al. (53) concluded that baPWV is associated with increased risk of total cardiovascular events and all-cause mortality, giving it potential for a universal clinical applicability. Although, according to (53), there is still a need to define reference values, expand data to non-Asian populations, validate path length estimation, and compare baPWV to cfPWV.

Additional peripheral PWV [carotid-radial PWV (crPWV) and femoral-ankle (faPWV)] have also been investigated (15, 23, 48). Because of the difference in structure of the peripheral and central arteries, faPWV and central PWV indexes correlate weakly (15). Furthermore, both crPWV and faPWV are not associated with risk factors of atherosclerosis (48) and present a limited increase (faPWV) or even decrease (crPWV) with age (6, 13).

Measuring foot-to-foot PWV presents some technical difficulties: the quality of the measurement depends, among other factors, on the skills of the operator, the characteristics of the subject, the accessibility of the arterial pulse, and the precision in the measurement of the length of the arterial path and of the transit time. Measurements are subjected to experimental errors and are seldom available both at the central and peripheral locations in the same subject.

Computational modeling of the hemodynamics provides an efficient tool to improve our understanding of pulse wave propagation in the arterial network. Models are based on physical principles that describe the blood flow and its interaction with the arterial wall. The morphology and structure of the arterial network are described through model parameters, whose values are known and prescribed. Physiological indexes, such as the PWV, can be computed over any path, as pressure and flow waveforms are available at every point of the arterial network.

One-dimensional (1D) models of the arterial hemodynamics have been largely used to simulate healthy and pathological conditions. Unlike three-dimensional models, 1D models are simple and fast computing, while efficient at predicting pressure and flow propagation along the arterial network. They have indeed been validated against in vitro (3, 9, 22, 29) and in vivo measurements in healthy (27, 35, 39), as well as in diseased arteries (42, 54).

Some studies have investigated the accuracy of PWV estimates using blood flow modeling. For instance, the Arteriograph PWV (Tensiomed, Budapest, Hungary), an estimate of the central stiffness obtained by occluding the brachial artery, was compared with the theoretical PWV and cfPWV in (49). The study highlighted that the Arteriograph PWV is rather an indicator of the brachial arteries stiffness than of the aorta. Modeling has also been used to assess the accuracy of different algorithms that compute PWV between two measurement sites (20) or local PWV at a single measurement site (2, 45). While these studies focus on a baseline model and include a few variations of the model parameters, they do not encompass the wide range of physiological variations present in a larger population.

The objective of this study is to assess, using 1D modeling, the accuracy of central and peripheral foot-to-foot PWV indexes at estimating the theoretical aortic stiffness. We compare indexes used in clinical practice and describe physical mechanisms underlying their correlations. To do so, we propose a novel methodology that creates a database of 3,325 virtual adult subjects by using a computational framework that combines multiple variations of cardiac and arterial properties within healthy ranges.

## METHODS

**Fig. 1.**
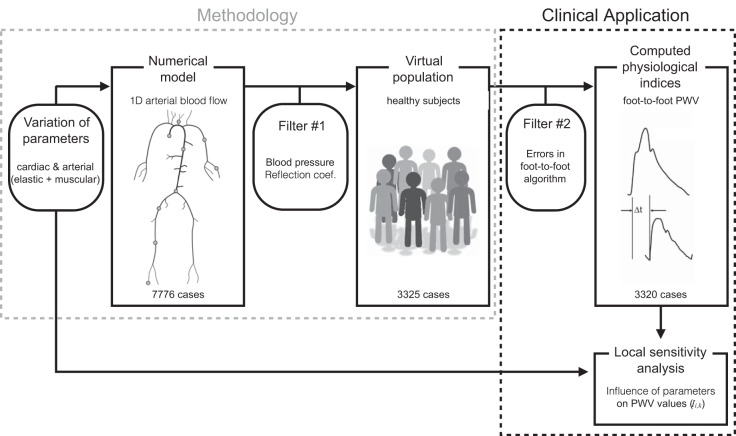
Our study consists of 2 parts: the development of a new methodology (i.e., the creation of a virtual population) and its clinical application [i.e., the assessment of foot-to-foot pulse wave velocity (PWV)]. By varying the cardiac and arterial parameters of the 1-dimensional (1D) model within healthy ranges, we create a set of 7,776 simulations. Rejection criteria (*filter #1*) are applied to eliminate nonphysiological data. Using the remaining 3,325 cases, we compute the physiological index of interest (i.e., foot-to-foot PWV) and reject 5 cases for which the PWV algorithm fails (*filter #2*). Peripheral and central PWV indexes are computed for each of the 3,320 cases using pressure waves measured at the dots in the Numerical model box. We also compute an index of local sensitivity analysis *Ī*_*i,k*_ that describes the effect of parameters variation on PWV values.

The methodology followed in this work is summarized in Fig. 1.

### Numerical Model of Pulse Wave Propagation

**Fig. A1.**
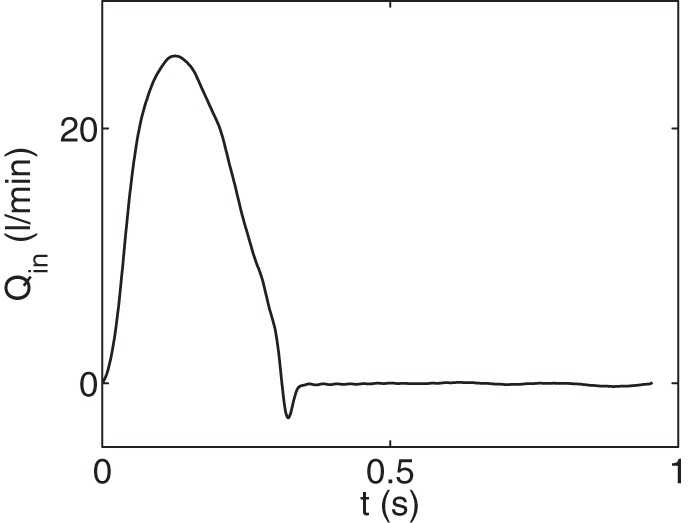
Inflow waveform *Q*_in_ prescribed at the aortic root of the baseline model: the heart rate is equal to 63 beats/min, the stroke volume to 83 ml, and the cardiac output is 5.2 l/min.

We used a nonlinear 1D model of blood flow in the 55 larger arteries of the human systemic circulation (4) (see box “Numerical model” in Fig. 1). Each artery of the network is characterized by its diameter *D*, length *L*, and arterial wall stiffness β. The arterial wall is assumed to be a thin elastic membrane. The peripheral branches of the 1D model are coupled to 0D three-element RCR Windkessel models that represent the resistive and compliant effects of the distal networks (arterioles and capillaries); each Windkessel is composed of two resistances (*R* = *R*_1_ + *R*_2_) and a compliance (*C*). At the aortic root, the flow *Q*_in_ measured in vivo in a healthy subject is prescribed as the inflow boundary condition in a reflective way (see Fig. A1 of appendix). Further details on the model and its parameters are displayed in the appendix.

### Generating a Virtual Database

In this study, we created a database of virtual healthy adult subjects. This was achieved by varying 7 significant parameters of the 55-artery model within ranges that are representative of a healthy population. Table 1 summarizes the incremental variations of the parameters considered in this study. These are the stiffness and diameter of elastic and muscular arteries, the peripheral vascular resistance, the heart rate (HR), and stroke volume (SV). The compliance of the peripheral circulation and the length of the arteries were not changed, as *1*) previous studies have shown that these parameters do not change the pressure and flow waveforms significantly (28, 54); and *2*) Sugawara et al. (44) showed that aortic tortuosity is mainly due to an elongation of the ascending aorta and has little impact on PWV measurements. As most of clinical studies in the literature describe arterial stiffness using estimates of local PWV rather than arterial elastic modulus *E*, the stiffness of the arterial wall was varied via the apparent PWV *c* (via the parameter *a* in *Eq. 11* of appendix).

**Table 1.** Relative variations of the seven parameters considered in this study, as a function of the age group

	Variation *v*, %						
	Age group, yr						
Parameter *p*	< 30	30–39	40–49	50–59	60–69	≥70	References
Elastic arteries PWV (*c*_el_)	−20	0	+30	+60	+90	+125	(36, 47)
Muscular arteries PWV (*c*_musc_)	−20	0	0	+15	+15	+30	(6, 37)
Elastic arteries diameter (*D*_el_)		[−10 0]*		+20	+20	+40	(1, 41)
Muscular arteries diameter (*D*_musc_)		[−10 0]*		+21	+21	+21	(1, 41)
Heart rate (HR)			[−15 0 +15]**				(15, 30)
Stroke volume (SV)			[−20 0 +20]**				(15, 30)
Peripheral vascular resistance (*R*)			[−10 0 +10]**				(40)

Variations are based on clinical observations and evolution with age within a healthy population. PWV, pulse wave velocity.

* Change in parameters resulting from the variability among subjects observed for age groups up to 49 yr.

** Change in parameters resulting from the variability among subjects observed for all age groups.

Because aging exerts opposing effects on central elastic large arteries and on distal muscular medium-sized arteries (12, 36), we considered different ranges of variation of the diameter and PWV for the elastic [from ascending to abdominal aorta (el)] and muscular arteries [all other peripheral arteries (musc)]. To avoid large structural discontinuities in upper limb arterial branches, the carotid artery was assumed to be a muscular artery, even if it presents structural properties similar to the aorta (12). We allowed for a large change in arterial stiffness in the elastic arteries (aorta), where a 2.4-fold increase of PWV can be observed over 60 yr (36, 47). On the contrary, a much smaller change with age is observed in muscular arteries, and hence, we only accounted for the variability of the PWV within a healthy population (6, 37). Elastic artery diameters were increased in larger proportion than muscular artery diameters to represent the artery dilation with age (1, 41). Additionally, a variation of ±10% was considered to represent muscular and elastic diameters variability within subjects (41). The heart variability was simulated through the flow waveform prescribed at the aortic root. Different HRs and SVs were prescribed, based on their relation to cardiac output (CO = HR·SV) and their variability in healthy subjects observed in clinical studies (15, 30). The systolic time (0.31 s) was kept constant in all inflows. The peripheral vascular resistance (*R*) was varied to represent the variability within a healthy population (40). All combinations of parameters were considered, and a total of 6·4^2^·3^4^ = 7,776 simulations were run.

Because the elastic modulus of the arterial wall is a function of the diameter and PWV (*Eq. 12* of appendix), we can compute its variation (*E*^*v*^) from its baseline value (*E*^0^):

(1) $$\frac{E^{v}}{E^{0}}={\left(\frac{a^{v}}{a^{0}}\right)}^{2}{\left(\frac{D^{v}}{D^{0}}\right)}^{-2b}$$

where the superscripts *v* and 0 refer respectively to a variation applied to a variable or to its baseline value. *Equation 1* follows from *Eq. 12* by assuming that the ratios of the time-varying (*D*^*v*^/*D*^0^), diastolic (*D*_d_^*v*^/*D*_d_^0^), and mean diameters (*D̄*^*v*^/*D̄*^0^) are all equal to *D*^*v*^/*D*^0^ and that the thickness is a constant fraction of the diameter for all cases. Since the diameters and PWV of the elastic arteries are varied by 4 and 6 levels respectively (Table 1), we obtained 4 × 6 = 24 distinct levels of variation of the elastic modulus of elastic arteries *E*_el_; these extend from −46 to +440%. Similarly, the elastic modulus of muscular arteries, *E*_musc_, presents 3 × 4 = 12 levels of variation, extending from −43 to +80%.

### Computed Physiological Indexes: Foot-to-Foot PWV

For each numerically converged simulation, we used the generated pressure waveforms to compute the following pulse wave velocities: the theoretical aortic PWV (aPWV_th_), the foot-to foot aortic PWV (aPWV), the foot-to-foot carotid-femoral PWV (cfPWV), the foot-to-foot brachial-ankle PWV (baPWV), the foot-to-foot femoral-ankle PWV (faPWV), and the foot-to foot carotid-radial PWV (crPWV).

The theoretical pulse wave velocity along the aorta (aPWV_th_) was calculated as the average of the theoretical wave speeds of all aortic segments, weighted by their lengths. The theoretical wave speed of each aortic segment (PWV_th,*i*_) was calculated as the integral of the wave speed along the artery length (*L*_*i*_) at the time of diastole (*t* = *t*_d_):

(2) $${\text{aPWV}}_{\text{th}}=\sum_{i}\frac{L_{i}{\text{PWV}}_{\text{th},i}}{L_{\text{ao}}}$$

(3) $${\text{PWV}}_{\text{th,}i}\left(t_{\text{d}}\right)=\frac{1}{L_{i}}\int_{0}^{L_{i}}\sqrt{\frac{\beta }{2\rho A_{\text{d}}}}A^{1/4}\left(x_{i},t_{\text{d}}\right)\text{d}x_{i}$$

where *L*_ao_ is the total length of the aorta, *x*_*i*_ is the axial coordinate along the segment *i*, ρ is the blood density, *A*(*x*, *t*) is the cross-sectional area of the lumen, *A*_d_(*x*) is the area at diastolic pressure, and *t*_d_ is the time when *A*(*x*_*i*_ = 0) is minimum. As detailed in the appendix (*Eq. 9*), β accounts for the material properties of the arterial wall. The integral was calculated using numerical quadrature.

Foot-to-foot PWV (PWV_ff_) were computed as Δ*L*/Δ*t*, with Δ*L* the distance traveled by the pulse wave, calculated as the difference between lengths of wave propagation from the heart

(4) $$\Delta L=\left\|L_{\text{heart-artery}1}-L_{\text{heart-artery2}}\right\|$$

and Δ*t* the transit time between the feet of the pressure waveforms. The aortic foot-to-foot PWV was computed between the aortic root and the aorto-iliac bifurcation. The measurement site at the ankle was taken at the distal point of the anterior tibial artery, while we considered the medial point of the carotid, iliac, brachial and radial arteries (see box “Numerical model” in Fig. 1). The feet of the pressure waveforms were detected using the clinical foot-to-foot algorithm detailed in Ref. 20 (intersection of the projection through the maximum gradient during systole and the horizontal through the minimum at diastole).

### Filter Criteria

Because all combinations of parameters in Table 1 were considered, we need to ensure that each numerical simulation converges and satisfies the following physiological conditions for a healthy population (*filter #1* in Fig. 1): *1*) the simulation converges within 11 complete cardiac cycles; *2*) the diastolic blood pressure (DBP) at the brachial artery is higher than 40 mmHg; *3*) the systolic blood pressure (SBP) at the brachial artery is lower than 200 mmHg; *4*) the pulse pressure (PP) at the brachial artery is higher than 25 mmHg and lower than 100 mmHg (19); and *5*) the reflection coefficient at the aorto-iliac bifurcation *R*_*f*_ is comprised between −0.3 and 0.3, as it is observed in young and old healthy subjects (21). *R*_*f*_ is obtained as follows: *R*_*f*_ = $\frac{Y_{\text{abd}}-Y_{\text{il}1}-Y_{\text{il}2}}{Y_{\text{abd}}+Y_{\text{il}1}+Y_{\text{il}2}}$, where *Y* = $\frac{A_{\text{d}}}{\rho c}$ is the characteristic admittance of the distal abdominal aorta (*Y*_abd_) and of the two proximal common iliac arteries (*Y*_il1_, *Y*_il2_) and *A*_d_ and *c* are the area and wave speed at diastole.

Furthermore, we excluded cases in which the foot-to-foot algorithm produced erroneous PWV (*filter #2* in Fig. 1). These cases were observed when the diastolic foot of the wave was not well detected (due to multiple local minima in diastole).

Out of the 7,776 cases defined, 3,320 simulations produced acceptable physiological results and were included in the PWV study. Out of the 4,456 excluded cases, 1,778 presented a low diastolic and/or high systolic pressure, 833 presented unphysiological pulse pressure, 1,188 (652) presented a reflection coefficient higher (lower) than 0.3 (−0.3), and 5 failed in the PWV algorithm.

### Local Sensitivity Analysis

The local sensitivity analysis allows to study the effects on PWV values of the variation of each parameter in Table 1. Based on the local sensitivity analyses in (27, 54), we defined the relative sensitivity index *I*_*i,k*_^*v*^ of output index PWV_*k*_ to the variation *v* of the model parameter *p*_*i*_ (*i* ∈ [1 : 7]) while the other six parameters *p*_*j*_ (*j* ≠ *i*) are kept constant:

(5) $$I_{i,k}^{v}=\left(\frac{{\text{PWV}}_{k}\left(p_{i}^{v},p_{j}\right)-{\text{PWV}}_{k}\left(p_{i}^{0},p_{j}\right)}{{\text{PWV}}_{k}\left(p_{i}^{0},p_{j}\right)}\right)\frac{1}{v}$$

where *p*_*i*_ is the parameter of interest at initial value (0%, *p*_*i*_^0^) and at increased or decreased value *v* (±*v*%, *p*_*i*_^*v*^), and *v* = $\frac{p_{i}^{v}-p_{i}^{0}}{p_{i}^{0}}$ is the variation of the parameter *p*_*i*_. For each of the seven input parameters *p*_*i*_, we then compute the average relative sensitivity index of output *k* for all variations *v* of one parameter *p*_*i*_ within all available physiological results:

(6) $${\overset{¯}{I}}_{i,k}=\underset{v}{\text{mean}}(I_{i,k}^{v})\;\text{for all parameters}\;p_{j}.$$

The average relative sensitivity index *Ī*_*i,k*_ represents an average percentage increase of output *k* for 1% increase in the input parameter *p*_*i*_.

### Statistical Analysis

Linear regression analysis and Pearson's correlation coefficient *r* were used to quantify correlations between PWV methods and indexes.

### Data Processing

We used our in-house code “Nektar” to solve the numerical model of pulse wave propagation (available on www.haemod.uk/Nektar.html). All 7,776 simulations were run on 8 core 16GB RAM machines using cloud computing (DigitalOcean.com). Signals were analyzed using customized Matlab software (The MathWorks, MA). The complete database of virtual subjects and computed indexes is available for download on the project webpage: www.haemod.uk/virtual-database.html.

## RESULTS

### General Characterization of the Virtual Database

**Fig. 2.**
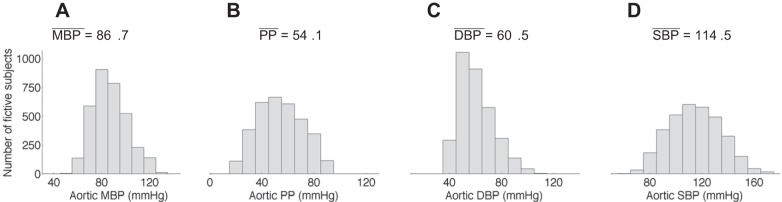
Distribution and mean value (in mmHg) of the mean blood pressure (MBP; *A*), pulse pressure (PP; *B*), and diastolic (DBP; *C*) and systolic (SBP; *D*) blood pressure at the aortic root for the virtual database. The DBP presents a distribution slightly truncated on the left, as a result of the filtering criteria (*filter #1*).

**Fig. 3.**
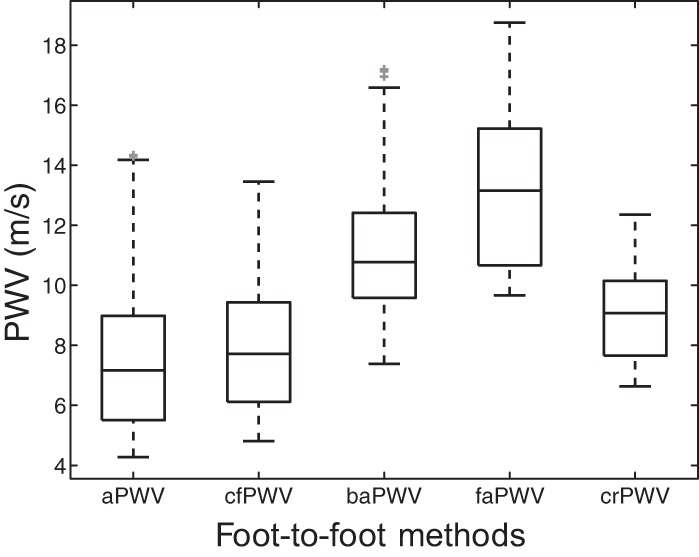
Distribution of central and peripheral foot-to-foot PWV of our virtual database. Each box indicates the 25th percentile, median, and 75th percentile; whiskers extend to minimum and maximum data points. Outliers are plotted individually in grey.

Blood pressures of all virtual subjects present physiological values with well-balanced distributions (Fig. 2). Cardiac outputs vary between 3.5 and 7.2 l/min, depending on the values of HR (53, 63, and 72 beats/min) and SV (66, 83, and 100 ml) prescribed. Figure 3 shows the distribution of central and peripheral foot-to-foot PWV. Central PWV (aPWV and cfPWV) have similar distributions with a median value ∼7.5 m/s, while peripheral PWV increase to higher distinct levels [median PWV at 10.8 m/s (baPWV), 13.1 m/s (faPWV) and 9.1 m/s (crPWV)]. Dispersions of PWV from the 25th to the 75th percentiles range from 2.5 to 3.5 m/s for all PWV, except for the faPWV (4.5 m/s). All PWV present normal distributions within physiological values, as observed in epidemiological studies of healthy subjects (19, 31, 47).

**Fig. 4.**
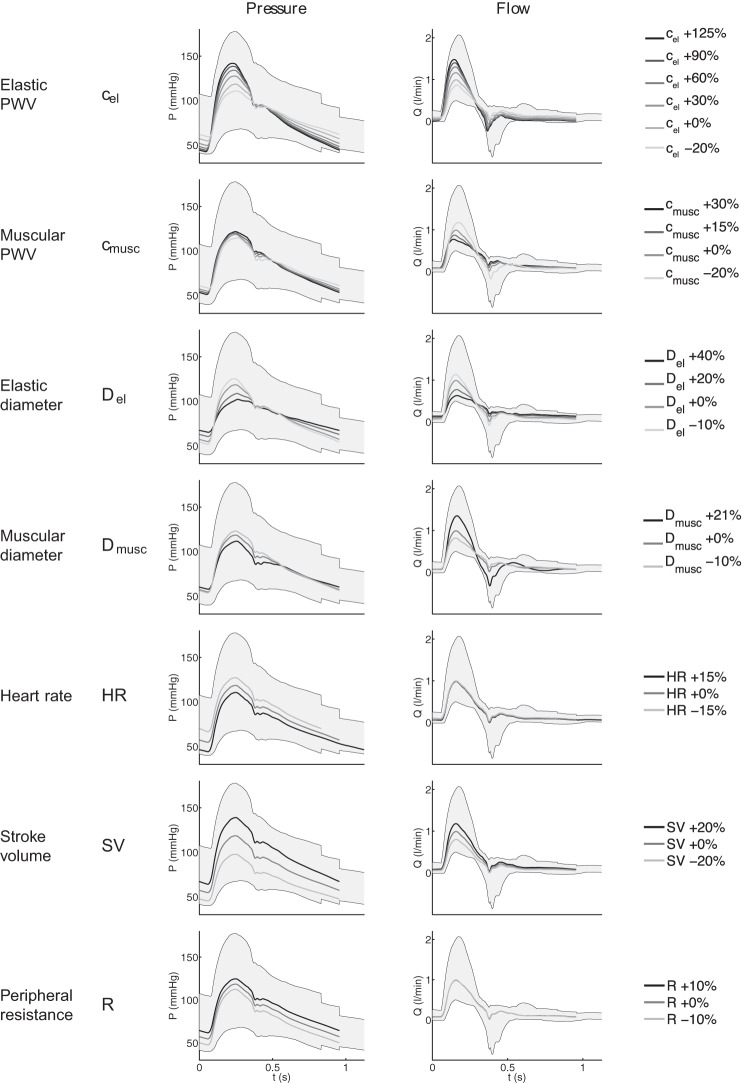
Variation of the brachial pressure (P; *left*) and flow (*Q*; *right*) waveforms induced by the variation of each parameter individually around the baseline (+ 0%). Parameters varied, from *top* to *bottom*: PWV of elastic arteries (*c*_el_) and of muscular arteries (*c*_musc_), diameter of elastic arteries (*D*_el_) and of muscular arteries (*D*_musc_), heart rate (HR), stroke volume (SV), and peripheral resistance (*R*). The gray shaded area represents the region covered by all superposed waveforms of the entire database.

There is a substantial change in the shape of pulse waveforms among the 3,325 virtual subjects. Figure 4 shows the individual effect of each varying parameter listed in Table 1 on the brachial pressure and flow waves around the model baseline. For pressure, increasing PWV or decreasing diameters amplifies the waveform, while decreasing HR or increasing SV or *R* shifts the pressure waveform up (Fig. 4, *left*). For the flow, muscular arterial parameters have opposite effects to elastic arterial parameters: decreasing muscular PWV or increasing muscular diameters amplifies the flow waveform, while increasing elastic PWV or decreasing elastic diameters has the same effect. Increasing SV amplifies the systolic flow waveform, while changing HR or *R* does not affect the flow (Fig. 4, *right*). Similar results were observed elsewhere in the arterial network.

**Fig. 5.**
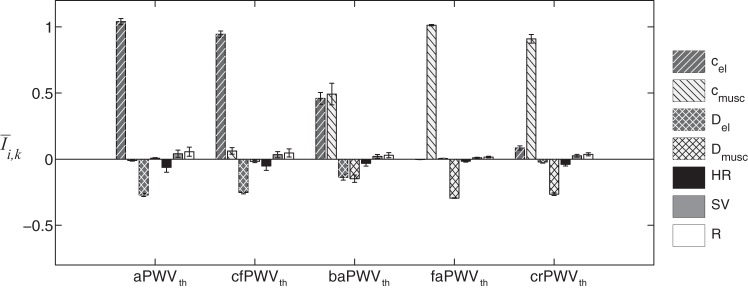
Averaged relative sensitivity indexes *Ī*_*i*,*k*_ of the 5 central and peripheral theoretical PWV (PWV_th_), as a function of the 7 input parameters indicated in the legend. Error bars represent the SD relative to each parameter around its output. a, Aortic; cf, carotid-femoral; ba, brachial-ankle; fa, femoral-ankle; cr, carotid-radial.

Changes in the seven varying parameters affect central and peripheral PWV values differently: Figure 5 presents the averaged relative sensitivity index *Ī*_*i,k*_ of the theoretical PWV_th_. As expected, the PWV parameters (*c*_el_ and *c*_musc_) induce large variations of the central and peripheral PWV: increasing *c*_el_ (*c*_musc_) leads to an increase of central (peripheral) PWV. Theoretical PWV indexes are relatively sensitive to the arterial diameters (*D*_el_ and *D*_musc_) which cause an opposite change in PWV values, as indicated by negative ${\overset{¯}{I}}_{D_{\text{el}},k}$ and ${\overset{¯}{I}}_{D_{\text{musc}},k}$. Finally, HR, SV, and *R* have a negligible effect on central and peripheral PWV, since their corresponding sensitivity indexes *Ī*_*HR,k*_, *Ī*_*SV,k*_, and *Ī*_*R,k*_ are, in absolute value, not larger than 7% for all PWV_th_.

### Comparison of the Foot-to-Foot PWV Against the Theoretical PWV in the Aorta

**Fig. 6.**
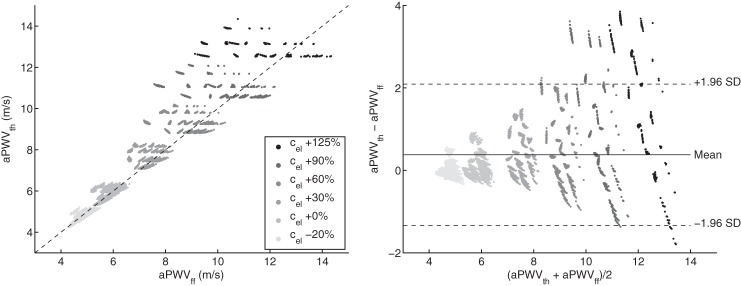
Theoretical PWV (aPWV_th_) vs. foot-to-foot PWV (aPWV_ff_) along the aorta (*left*) and corresponding Bland-Altman plot (*right*). Each dot represents one of the 3,320 virtual subjects. Gray levels indicate the variation from baseline of the prescribed elastic arteries PWV (*c*_el_): from −20% (light gray) to +125% (black). *Left*: the dashed line indicates identity. *Right*: the continuous line indicates the mean value and the dashed lines indicate mean ± 1.96 SD.

Figure 6 compares the foot-to-foot (aPWV_ff_) and the theoretical (aPWV_th_) pulse wave velocities along the aorta. Both aPWV_th_ and aPWV_ff_ vary from 4.2 to 14.3 m/s. aPWV_ff_ correlates well with aPWV_th_ (*r* = 0.946), although aPWV_ff_ tends to underestimate aPWV_th_ on average (difference: 0.38 ± 0.87 m/s). This deviation increases with higher elastic artery stiffness, as shown by the Bland-Altman analysis (Fig. 6, *right*).

**Fig. 7.**
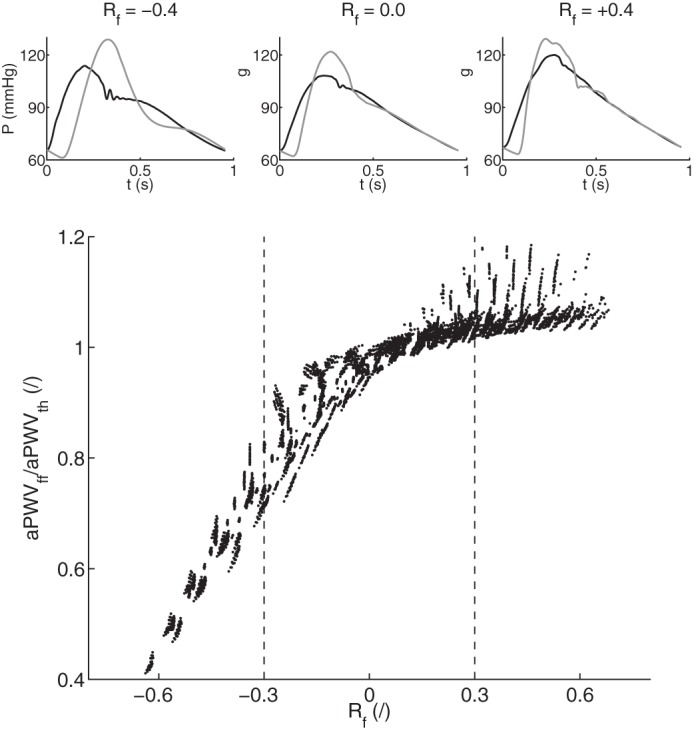
*Bottom*: deviation of the ratio of PWV methods along the aorta (foot-to-foot aPWV_ff_ over theoretical aPWV_th_) as a function of the reflection coefficient *R*_*f*_ at the aorto-iliac bifurcation for all 3,320 + 1,840 converging cases. *Top*: examples of aortic (black) and iliac bifurcation (gray) pressure waveforms for 3 values of the reflection coefficient *R*_*f*_: outside (*R*_*f*_ = ±0.4) and inside (*R*_*f*_ = 0.0) the physiological limit.

Figure 7, *bottom*, presents the ratio of aPWV_ff_ to aPWV_th_ as a function of the reflection coefficient *R*_*f*_ at the aorto-iliac bifurcation, for all converging cases (i.e., 3,320 + 1,840 cases, including *R*_*f*_ > 0.3 and *R*_*f*_ < −0.3). The foot-to-foot PWV deviates from the theoretical value from 20 to 50% if *R*_*f*_ < −0.3, while the deviation is smaller than 5% if *R*_*f*_ > 0.3. Pressure waveforms in Fig. 7, *top*, illustrate the change in wave shape for different reflection coefficients. The pressure waveform at the iliac bifurcation presents oscillations if *R*_*f*_ is outside the physiological range: during the diastolic decay if *R*_*f*_ < −0.3 and in late systole if *R*_*f*_ > 0.3.

**Fig. 8.**
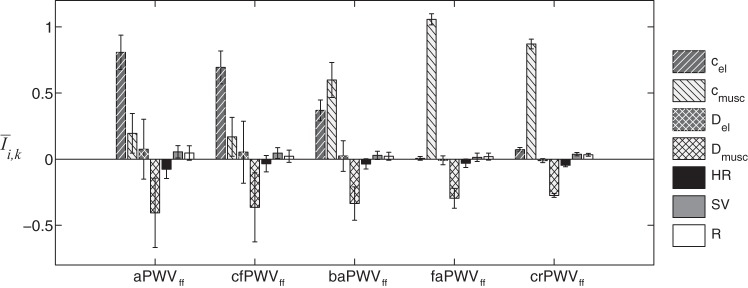
Averaged relative sensitivity indexes *Ī*_*i*,*k*_ of the 5 central and peripheral foot-to-foot PWV (PWV_ff_), as a function of the 7 input parameters indicated in the legend. Error bars represent the SD relative to each parameter around its output.

The deviation of foot-to-foot PWV from theoretical PWV can also be observed in the sensitivity analysis of PWV_ff_ to the seven varying parameters (Fig. 8). Unlike the sensitivity analysis of theoretical PWV (Fig. 5), the influence of the diameter of muscular arteries is much larger for central PWV. We also observe a high dispersion of the sensitivity indexes around the mean value, as shown by the considerable standard deviations of average indexes *Ī*_*i,k*_.

### Comparison Between Foot-to-Foot PWV

**Fig. 9.**
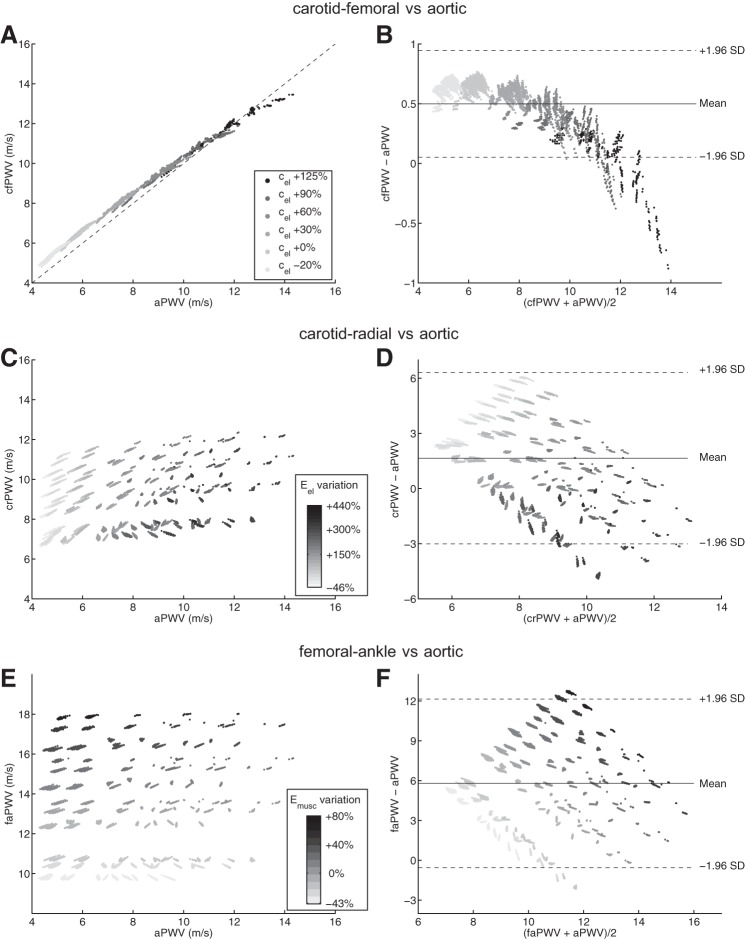
Comparison between foot-to-foot PWV and aortic PWV (aPWV; *left*) and corresponding Bland-Altman plots (*right*). Each dot represents one virtual subject from the 3,320 physiological cases of our database. cfPWV for the 6 levels of *c*_el_ considered (*A* and *B*), crPWV for the 24 levels of *E*_el_ considered (*C* and *D*), and faPWV for the 12 levels of *E*_musc_ considered (*E* and *F*). In Bland-Altman plots, the continuous line indicates the mean value, and dashed lines indicate mean ± 1.96 SD. In *A*, the dashed line indicates identity.

**Fig. 10.**
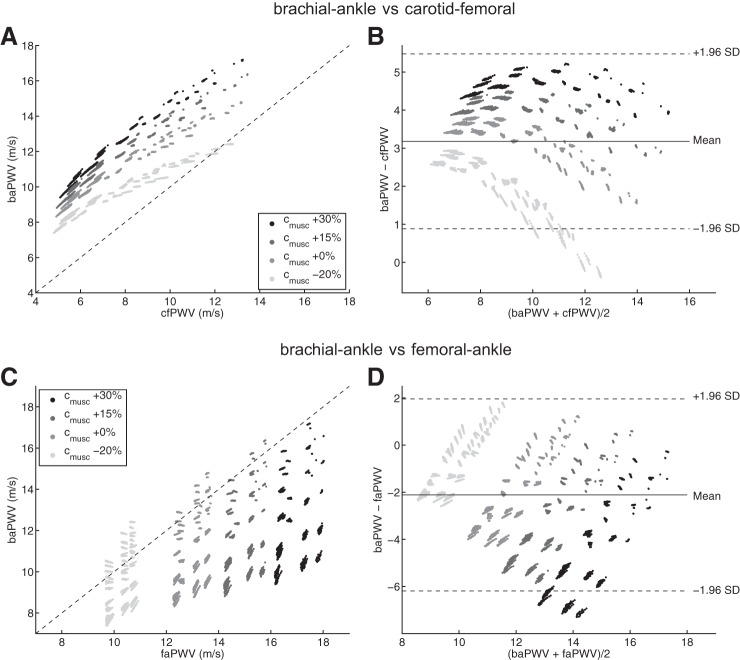
Comparison between peripheral and central foot-to-foot pulse wave velocities (*left*) and corresponding Bland-Altman plots (*right*). Each dot represents one virtual subject from the 3,320 physiological cases of our database. baPWV vs. cfPWV (*A* and *B*) and baPWV vs. faPWV (*C* and *D*) for the 4 levels of *c*_musc_ considered. In Bland-Altman plots, the continuous line indicates the mean value, dashed lines indicate mean ± 1.96 SD. In *A* and *C*, the dashed line indicates identity.

This section compares central and peripheral foot-to-foot PWV indexes. We focus on *1*) how central and peripheral PWV indexes are related to aortic PWV (Fig. 9), and *2*) on correlations often used in the clinic, involving cfPWV, baPWV, and faPWV (Fig. 10).

#### Comparison with the aortic PWV.

Both central PWV indexes (cfPWV and aPWV) increase with the elastic arteries PWV parameter *c*_el_ (Fig. 9, *A* and *B*). There is a strong correlation between cfPWV and aPWV (*r* = 0.998). However, the carotid-femoral PWV slightly overestimates the aortic PWV (mean difference: 0.50 ± 0.23 m/s), as shown by the Bland-Altman plot. With increased stiffening of the aorta (PWV larger than 12 m/s), this trend reverses and cfPWV underestimates aPWV.

While the aortic PWV increases largely with *E*_el_, the peripheral crPWV does not (Fig. 9, *C* and *D*). Instead of elastic arteries properties, the factors influencing crPWV are muscular arteries properties, as shown by the relative sensitivity index *Ī*_*i,k*_ of crPWV (Figs. 5 and 8). Similarly, the femoral-ankle PWV is mainly influenced by muscular properties of arteries: it raises with increasing elastic modulus of muscular arteries *E*_musc_ (Fig. 9, *E* and *F*).

#### Clinical correlations: baPWV vs. cfPWV and faPWV.

Brachial-ankle PWV correlates well with cfPWV (*r* = 0.829) although it overestimates it (mean difference: 3.18 ± 1.17 m/s; Fig. 10, *A* and *B*). This overestimation is strongly related to the stiffness of muscular arteries (*c*_musc_): individual correlations for each level of *c*_musc_ increase up to *r* = 0.991. The parameter *c*_musc_ is also (together with the parameter *D*_musc_) one of the main factors that determine the value of the peripheral femoral-ankle PWV, as faPWV increases with *c*_musc_ (Fig. 10, *C* and *D*). These figures also show a weak correlation between faPWV and baPWV (*r* = 0.566) as baPWV presents low to high values (from 9 to 17 m/s) if faPWV >16 m/s.

### Influence of the Aortic Elastic Modulus on PWV

**Fig. 11.**
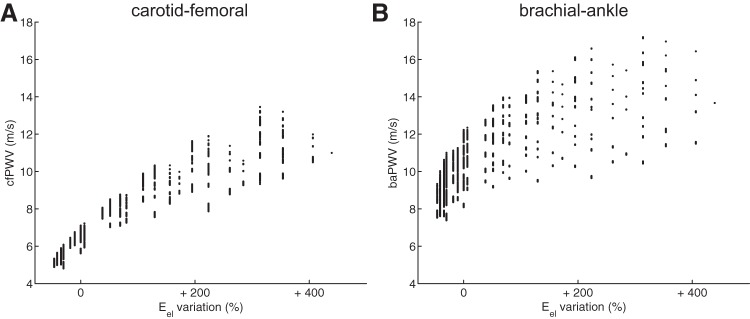
Relation between foot-to-foot PWV and the variation of the elastic modulus of elastic arteries (*E*_el_); carotid-femoral PWV (cfPWV; *A*) and the brachial-ankle PWV (baPWV; *B*). Each dot represents 1 virtual subject from the database.

Figure 11 relates the elastic modulus of elastic arteries (*E*_el_) to both cfPWV (*A*) and baPWV (*B*). We observe a distinct increase in cfPWV with increasing *E*_el_. Despite baPWV also raising with increasing *E*_el_, we observe more dispersion of the values of this peripheral index.

## DISCUSSION

### Assessment of Foot-to-Foot PWV Indexes

The distributions of central and peripheral PWV obtained from our virtual population are in agreement with clinical observations (15): central PWV varies between 6 and 9 m/s and peripheral PWV between 10 and 15 m/s in 50% of the cases (Fig. 3). Using this population we have investigated how central PWV and peripheral foot-to-foot PWV are related to each other and to the theoretical aortic stiffness. In the following, we discuss the main clinically relevant results of our study: *1*) cfPWV is a good indicator of aortic stiffness, *2*) baPWV is influenced by both central and peripheral arterial properties, and *3*) muscular crPWV and faPWV indexes do not quantify aortic stiffness.

The elastic modulus of the aorta is well estimated by the central cfPWV (Fig. 11*A*), and there is a strong correlation between cfPWV and aPWV (Fig. 9, *A* and *B*): cfPWV differs from aPWV by <10%. These observations confirm the choice of cfPWV as the gold standard index for the measurement of aortic stiffness (26). On the contrary, baPWV does not present a direct relation with *E*_el_ (Fig. 11*B*). This index is indeed influenced by the mechanical properties of both central and peripheral arteries, as shown by the sensitivity index *Ī*_*i,k*_ of baPWV (Fig. 5) and confirmed by the results in Figs. 10: the larger the stiffness of muscular arteries (*c*_musc_), the more baPWV overestimates cfPWV. These observations agree with clinical conclusions from the population study of Choo et al. (15): cfPWV is influenced by properties of central arterial stiffness while baPWV is affected by mixed properties of both central and peripheral arterial stiffness.

Muscular crPWV and faPWV indexes are not good substitutes for central PWV, as they do not vary with different levels of aortic stiffness levels (Figs. 9, *C–F*). In fact, the paths traveled by pulse waves measured in crPWV or faPWV do not include the aorta. This result is in line with the clinical observation from Tillin et al. (48) that muscular arteries PWV are poor indicators of arterial stiffening.

Foot-to-foot PWV were computed from pressure waveforms [those are widely used by clinical devices (26)] using the algorithm described in Gaddum et al. (20). This algorithm is used in clinical practice and is, to our knowledge, the most efficient algorithm for computing foot-to-foot PWV. Foot-to-foot aortic PWV correlates relatively well with the theoretical PWV (Fig. 6), though the foot-to-foot index underestimates the theoretical value by ∼10% in normal arteries and by up to 30% in stiffer arteries; similar results have been observed in the studies of Trachet et al. (49) and Gaddum et al. (20). Differences between sensitivity analyses of theoretical and foot-to-foot PWV (Figs. 5 and 8) are also explained by this bias in the foot-to-foot PWV method. As shown in Fig. 7, the bias is caused by wave reflections at the discontinuity between the aorta and leg arteries, which induce oscillations in the aortic pressure wave at the end of diastole (when the foot of the wave occurs). These oscillations indicate the presence of reflected waves, which have an adverse effect on PWV estimated by the foot-to-foot method. A similar adverse impact of wave reflections on PWV estimates has been previously reported for the loop and single-point methods (2, 11). Therefore, the foot-to-foot technique should be used carefully in the clinic, as it is not a surrogate for the reflection-free theoretical PWV.

### Creation of a Database of Virtual Subjects

The database of virtual subjects was generated by varying arterial and cardiac parameters of the blood flow model by a range of physiological values for healthy subjects taken from the literature. Similar ranges were used in the study of Trachet et al. (49) for the elasticity of the vessel wall, the cardiac parameters and peripheral resistance. However, our study also looked at the effect of distinguishing among properties of elastic and muscular arteries, changing arterial diameters, and varying all parameters simultaneously.

Changes in the elasticity of vessels can be achieved through the variation of the arterial elastic modulus *E*. Given that measures of *E* are scarce in clinical literature, we enforced variations of the local PWV *c*, which is related to *E* and to the diameter *D* (*Eq. 12* of the appendix). Therefore, a change in *D* also modifies the PWV parameter. This observation is also valid for other stiffness indexes (e.g., area compliance and distensibility).

While we used several values for elastic and muscular artery parameters, we did not enforce a relation between their variations. This led to large discontinuities at the aorto-iliac bifurcation and along the aorta and to the creation of nonphysiological reflected waves. Through the computation of the reflection coefficient at the aorta-iliac bifurcation (*filter #1* in Fig. 1), these nonphysiological combinations of parameters were identified and excluded.

### Limitations

We used a nonlinear 1D model of the arterial hemodynamics to generate the database. Results presented in this study depend on the following characteristics of the model: *1*) the tube law, *2*) the type of fluid viscous dissipation, *3*) the boundary conditions, and *4*) the arterial network definition. The following choices have been made to get a good compromise between computing time and accuracy.

*1*) We considered an elastic tube law, whose mechanical properties are independent of arterial blood pressure. Clinical studies show, however, that the stiffness of the arterial wall raises with increasing blood pressure (34). This behavior can be simulated using a nonlinear tube law (38) or more complex structure-based constitutive laws (50). At the expense of an increase in the computational cost, another improvement is the use of a visco-elastic model of the arterial wall which may have a considerable effect on waveforms (3), particularly in hypertensive subjects (5).

*2*) Our model of fluid viscous dissipation is based on a prescribed velocity profile. This choice gives satisfactory results in central arteries but might not be as accurate in peripheral arteries, where viscous dissipation is greater (14). Our current model could be improved with the evaluation of a time- and space-dependent velocity profile throughout the network (8), providing a more precise approximation of the wall friction term.

*3*) A physiological flow waveform was prescribed as inlet boundary condition (Fig. A1). Through variations in the amplitude and duration of this wave, we were able to model physiological changes in HR, SV, and cardiac output. This inflow boundary condition could be improved by coupling the 1D network to a 0D model of the heart (18, 33, 38) to provide a more physiological description of the cardiac-arterial coupling.

*4*) Our model is made of the 55 larger systemic arteries; it does not include neither hand arteries nor a detailed cerebral arterial tree. However, this arterial network is suitable for our particular application as it contains all distal arteries where pulse waves measurements are usually taken to calculate foot-to-foot PWV indexes.

Despite these limitations, our model was able to produce a database of virtual subjects with hemodynamic properties and pulse waveforms which are realistic under normal physiological conditions.

### Perspectives

Our database of virtual subjects could provide valuable insight into hemodynamic mechanisms that are important for the design of large cohort clinical studies and for analyzing their results. Our methodology allows to describe physical mechanisms underlying correlations observed in the clinic. This is extremely difficult in practice due to measurement errors, the difficulty in taking simultaneous measurements at all vessels of interest, and the inability to isolate variations in physical parameters without compensatory effects of cardiovascular homeostatic reflexes.

Additional cardiovascular indexes calculated via pulse-wave analysis could be assessed using our virtual population. For example, the stiffness index (17), the Arteriograph PWV (49), the augmentation index (24), pulse pressure amplification (7), and ankle-brachial index (16) could also be assessed theoretically and related to other physiological indexes. Besides indexes, the virtual database could serve to test new algorithms derived from pulse wave analysis, such as methods for estimating central blood pressure from peripheral measurements (32).

In future works, we plan to expand the current database by including virtual subjects with pathology that can be simulated using 1D modeling (e.g., hypertension, diabetes).

### Conclusion

We have presented a new methodology for the theoretical assessment of computed physiological indexes and algorithms based on pulse wave analysis. The methodology consists of a database of virtual arterial waveforms that we have created using a 1D numerical model of blood flow in the 55 larger systemic arteries. The set of 3,325 virtual subjects encloses a wide selection of possible cases under normal physiological conditions that could be encountered in a clinical study. Furthermore, we have provided several postprocessing tools to quantify the effect of changes in cardiovascular parameters on the computed index.

Using our virtual population, we have assessed the accuracy of central and peripheral foot-to-foot PWV to quantify aortic stiffness. Our study confirms clinical observations: cfPWV is a good indicator of aortic stiffness, muscular PWV (crPWV and faPWV) are poorly related to cfPWV, and the baPWV foot-to-foot index is influenced by both central and peripheral arterial properties. Lastly, we have noted that the foot-to-foot PWV method is sensitive to the presence of reflected waves during late diastole, which introduce errors in the PWV estimates.

## GRANTS

M. Willemet and J. Alastruey gratefully acknowledge the support of an Engineering and Physical Sciences Research Council (EPSRC) Project Grant EP/K031546/1 and the Centre of Excellence in Medical Engineering funded by the Wellcome Trust and EPSRC Grant WT 088641/Z/09/Z. J. Alastruey gratefully acknowledges the support of a British Heart Foundation Intermediate Basic Science Research Fellowship (FS/09/030/27812).

## DISCLOSURES

No conflicts of interest, financial or otherwise, are declared by the author(s).

## AUTHOR CONTRIBUTIONS

Author contributions: M.W. conception and design of research; M.W. performed experiments; M.W. analyzed data; M.W., P.C., and J.A. interpreted results of experiments; M.W. prepared figures; M.W. drafted manuscript; M.W. and J.A. edited and revised manuscript; M.W., P.C., and J.A. approved final version of manuscript.

## Appendix

### The Numerical Model

We used a nonlinear 1D model of blood flow in compliant arteries. The 1D governing equations are based on the Euler equations of conservation of mass and momentum. These assume blood to be an incompressible and Newtonian fluid:

(7a) $$\frac{\partial A}{\partial t}+\frac{\partial \left(AU\right)}{\partial x}=0,$$

(7b) $$\frac{\partial U}{\partial t}+U\frac{\partial U}{\partial x}=-\frac{1}{\rho }\frac{\partial P}{\partial x}-\frac{22\pi \mu U}{\rho A},$$

where *x* is the axial coordinate along the vessel, *t* is the time, *A*(*x*, *t*) is the cross-sectional area of the lumen, *U*(*x*, *t*) is the axial blood flow velocity averaged over the cross-section, *P*(*x*, *t*) is the blood pressure averaged over the cross section, and ρ = 1,050 kg/m^3^ and μ = 2 mPa s are, respectively, the constant density and viscosity of blood. Energy losses were neglected at bifurcations, and we further assumed a velocity profile close to plug flow in all arteries. These equations were coupled with a pressure-area relation (tube law) that describes the arterial wall as a thin, elastic, homogeneous and incompressible membrane:

(8) $$P=P_{\text{d}}+\frac{\beta }{A_{\text{d}}}\left(\sqrt{A}-\sqrt{A_{\text{d}}}\right),$$

with *A*_d_(*x*) the luminal area at diastolic pressure *P*_d_. β(*x*) Accounts for the material properties of the arterial wall of elastic modulus *E*(*x*) and thickness *h*(*x*) through

(9) $$\beta \left(x\right)=\frac{4}{3}\sqrt{\pi }Eh.$$

Following the definition of the local PWV *c* = $\sqrt{\frac{A}{\rho }\frac{\partial P}{\partial A}}$, the parameter β is related to *c* for our particular choice of pressure-area law:

(10) $$\beta =\frac{2\rho c^{2}A_{\text{d}}}{\sqrt{A}}.$$

Additionally, *c* is related to the artery size. Reymond et al. (39) observed that there is a general trend of an inverse global relation between artery mean diameter *D̄* and *c*, for large arteries with a lumen diameter >5 mm. They proposed the empirical inverse power curve fitting:

(11) $$c=\frac{a}{{\left(\overset{¯}{D}\right)}^{b}},$$

where the coefficient *b* is fixed to 0.3 and the coefficients *a* are listed in Table A1. With the additional assumption that the arterial wall thickness is proportional to the diastolic diameter (34), *h* = α*D*_d_ [typically α = 7%, cf Table 1 in Caro et al. (14)], the elastic modulus *E* of the arterial wall can then be related to *c* and to the time-varying [*D*(*x*)], diastolic (*D*_d_), and mean (*D̄*) diameters of the artery using *Eqs. 9*-*11*:

(12) $$E=\frac{3}{4}\frac{\rho }{h}\frac{{\left(D_{\text{d}}\right)}^{2}}{D}c^{2}=\frac{3}{4}\frac{\rho }{\alpha }\frac{D_{\text{d}}}{D}\frac{a^{2}}{{\left(\overset{¯}{D}\right)}^{2b}}.$$

**Table A1.** Parameters of the arterial tree of the baseline model

			PWV			
Arterial Segment	Length (*L*), cm	Diameter (*D*_in_ → *D*_out_) mm	*a*	*c*_in_ → *c*_out_, m/s	Peripheral Resistance *R*, 10^10^ Pa·s·m^−3^	Peripheral Compliance *C*, 10^−10^ m^3^/Pa
1. Ascending aorta	5.8	27.6 → 27.4	14.3	5.18 → 5.19	—	—
2. Aortic arch A	2.3	23.7 → 22.7	14.3	5.43 → 5.5	—	—
3. Brachiocephalic	3.9	19.3 → 17.3	14.3	5.79 → 5.99	—	—
4. R. subclavian	3.9	11.2 → 8.85	14.3	6.84 → 7.36	—	—
5. R. common carotid	10.8	10.7 → 5.66	14.3	6.94 → 8.44	—	—
6. R. vertebral	17.1	3.72 → 2.82	15.6	10.5 → 11.4	0.451	0.902
7. R. brachial	48.5	8.04 → 4.7	15.6	8.28 → 9.75	—	—
8. R. radial	27	3.72 → 3.11	15.6	10.5 → 11	0.396	0.987
9. R. ulnar A	7.7	3.72 → 3.42	15.6	10.5 → 10.7	—	—
10. R. interosseous	9.1	2.12 → 1.82	15.6	12.4 → 13	6.32	0.325
11. R. ulnar B	19.7	3.22 → 2.81	15.6	10.9 → 11.4	0.396	0.769
12. R. internal carotid	20.5	5.7 → 4.31	15.6	9.2 → 10.0	0.188	2.58
13. R. external carotid	18.7	2.53 → 1.52	15.6	11.8 → 13.7	1.04	1.93
14. Aortic arch B	4.5	20.4 → 19.8	14.3	5.69 → 5.74	—	—
15. L. common carotid	16	9.56 → 4.85	14.3	7.19 → 8.84	—	—
16. L. internal carotid	20.5	4.32 → 3.34	15.6	10 → 10.8	0.188	1.89
17. L. external carotid	18.7	1.94 → 1.23	15.6	12.8 → 14.6	1.04	1.73
18. Thoracic aorta A	6	19.1 → 18.1	14.3	5.81 → 5.91	—	—
19. L. subclavian	3.9	10.7 → 8.37	14.3	6.93 → 7.49	—	—
20. L. vertebral	17	3.72 → 2.82	15.6	10.5 → 11.4	0.451	0.902
21. L. brachial	48.5	8.04 → 4.7	15.6	8.28 → 9.75	—	—
22. L. radial	27	3.52 → 2.82	15.6	10.6 → 11.4	0.396	0.848
23. L. ulnar A	7.7	4.31 → 4.31	15.6	10.0 → 10.0	—	—
24. L. interosseous	9.1	1.82 → 1.82	15.6	13.0 → 13.0	6.32	0.277
25. L. ulnar B	19.7	4.11 → 3.71	15.6	10.2 → 10.5	0.396	1.3
26. Intercostals	9.2	12.2 → 9.31	14.3	6.66 → 7.24	0.6	10.4
27. Thoracic aorta B	12	15.9 → 12.5	14.3	6.15 → 6.62	—	—
28. Abdominal aorta A	6.1	11.8 → 11.8	14.3	6.73 → 6.73	—	—
29. Celiac A	2.3	7.68 → 6.82	14.3	7.68 → 7.97	—	—
30. Celiac B	2.3	5.17 → 4.88	14.3	8.67 → 8.83	—	—
31. Hepatic	7.6	5.4 → 4.41	15.6	9.35 → 9.94	0.272	2.05
32. Gastric	8.2	3.22 → 3.02	15.6	10.9 → 11.1	0.406	0.821
33. Splenic	7.2	4.21 → 3.91	15.6	10.1 → 10.3	0.174	1.4
34. Superior mesenteric	6.8	7.77 → 7	14.3	7.65 → 7.9	0.0698	4.81
35. Abdominal aorta B	2.3	11.2 → 11	14.3	6.85 → 6.88	—	—
36. L. renal	3.7	5.17 → 5.16	14.3	8.67 → 8.67	0.0848	2.31
37. Abdominal aorta C	2.3	11.5 → 11.5	14.3	6.79 → 6.79	—	—
38. R. renal	3.7	5.17 → 5.16	14.3	8.67 → 8.67	0.0848	2.31
39. Abdominal aorta D	12.2	11.3 → 10.7	14.3	6.83 → 6.94	—	—
40. Inferior mesenteric	5.8	4.7 → 3.21	15.6	9.74 → 10.9	0.516	1.33
41. Abdominal aorta E	2.3	10.5 → 10.1	14.3	6.97 → 7.05	—	—
42. L. common iliac	6.8	7.9 → 7.01	18.0	9.63 → 9.99	—	—
43. R. common iliac	6.8	7.9 → 7.01	18.0	9.63 → 9.99	—	—
44. L. external iliac	16.6	6.42 → 6.12	18.0	10.3 → 10.4	—	—
45. L. internal iliac	5.8	4.05 → 4.05	19.7	12.9 → 12.9	0.596	1.37
46. L. femoral	50.9	5.25 → 3.85	19.7	11.9 → 13.1	—	—
47. L. deep femoral	14.5	4.05 → 3.75	19.7	12.9 → 13.2	0.358	1.27
48. L. posterior tibial	36.9	3.14 → 2.84	19.7	13.9 → 14.3	1.06	0.743
49. L. anterior tibial	39.8	2.64 → 2.33	19.7	14.6 → 15.2	1.06	0.513
50. R. external iliac	16.6	6.42 → 6.12	18.0	10.3 → 10.4	—	—
51. R. internal iliac	5.8	4.05 → 4.05	19.7	12.9 → 12.9	0.596	1.37
52. R. femoral	50.9	5.25 → 3.85	19.7	11.9 → 13.1	—	—
53. R. deep femoral	14.5	4.05 → 3.75	19.7	12.9 → 13.2	0.358	1.27
54. R. posterior tibial	36.9	3.14 → 2.84	19.7	13.9 → 14.3	1.06	0.743
55. R. anterior tibial	39.8	2.64 → 2.33	19.7	14.6 → 15.2	1.06	0.513

Arterial length *L*, mean cross-sectional diameter *D*, pulse wave velocity coefficient *a* (in *Eq. 11*) and value *c* at diastolic pressure *P*_d_, total peripheral resistance *R* = *R*_1_ + *R*_2_, and peripheral compliance *C*. The subscripts in and out refer to the inlet and outlet of the arterial segment. R., right; L. left.

The 1D model used in this study consists of the 55 larger systemic arteries (cf. box “Numerical model” in Fig. 1). Each artery is modeled as a linearly tapered vessel. The in vivo inflow wave prescribed at the aortic root, *Q*_in_, is shown in Fig. A1. Terminal vessels are coupled to 0D windkessel models made of two resistances in series (*R*_1_ and *R*_2_) and a compliance (*C*); the outflow pressure to the Windkessel model is set to *P*_out_ = 10 mmHg. The value of the first resistance is set equal to the characteristic impedance *Z*_*c*_ of the end point of the 1D vessel to reduce spurious reflections (4). Table A1 presents the parameters of the baseline 1D model.

Initial conditions are (*A*, *U*, *P*) = (*A*_0_, 0, 0) in all segments, with *A*_0_ the area that yields *A*_d_ at *P* = *P*_d_ = 95 mmHg. Each simulation is run for at least 11 cardiac cycles to ensure that a periodic state is reached. The system of *Eqs. 7* and *8* is solved numerically using a high order discontinuous Galerkin method with a spectral/hp spatial discretization (of polynomial order 3) and a second-order Adams-Bashfort time integration scheme. Each simulation runs in ∼6 min on a standard computer (single CPU). We refer the reader to Alastruey et al. (4) for further details regarding the model and its numerical resolution.
